# Postharvest Dynamics of Photosynthesis in Fresh‐Cut Lettuce

**DOI:** 10.1111/ppl.70433

**Published:** 2025-08-03

**Authors:** Priscille Steensma, Alexey Shapiguzov, Leevi Annala, Julia P. Vainonen, Kirsi S. Mikkonen, Saijaliisa Kangasjärvi

**Affiliations:** ^1^ Department of Food and Nutrition University of Helsinki Helsinki Finland; ^2^ Production Systems Natural Resources Institute Finland (Luke) Turku Finland; ^3^ Organismal and Evolutionary Biology Research Programme, Faculty of Biological and Environmental Sciences University of Helsinki Helsinki Finland; ^4^ Viikki Plant Science Centre University of Helsinki Helsinki Finland; ^5^ Helsinki Institute of Sustainability Science (HELSUS) University of Helsinki Helsinki Finland; ^6^ Department of Agricultural Sciences, Faculty of Agriculture and Forestry University of Helsinki Helsinki Finland

**Keywords:** chlorophyll fluorescence, lettuce, modified atmosphere packaging, NPQ, regulations of photosynthesis

## Abstract

Increasing the availability of fresh vegetables and reducing food waste are essential for healthy and sustainable production. However, fresh‐cut vegetables such as lettuce ( *Lactuca sativa* L.) often experience rapid quality loss after harvest and processing. To maintain freshness in retail, modified atmosphere packaging (MAP) with low oxygen (O_2_) and high carbon dioxide (CO_2_) concentrations, combined with refrigeration, is commonly used. Packaged leaves are then displayed under dark or low‐light conditions. The postharvest physiology of lettuce under these conditions (including changes in energy metabolism, photosynthesis, senescence progression, and light‐dependent metabolic alterations) remains poorly understood, limiting the development of strategies to extend shelf life. Using spectroscopic and biochemical approaches, we investigated the physiological changes in fresh‐cut lettuce stored in MAP under refrigeration in either darkness or constant low light. Our analysis revealed distinct light‐dependent and light‐independent adjustments in photosynthesis. MAP triggered rapid and dramatic changes in photosynthetic light reactions, detectable within 1 h of packaging, as observed by pulse amplitude modulated (PAM) and OJIP kinetics assays. These changes were likely associated with compromised electron sink strength in the photosynthetic electron transfer chain and altered dynamics of the energy component of nonphotochemical quenching (qE‐NPQ). Notably, these functional alterations coincided with only minor modifications in photosynthetic supercomplexes, as determined by blue native gel electrophoresis. The MAP‐induced changes in photosynthesis deteriorated during storage in darkness but were reversed by storage under low light, likely due to photosynthetic gas exchange. Our findings provide new insights into photosynthesis of packaged lettuce and highlight promising physiological readouts for assessing lettuce quality in retail settings.

## 1 Introduction

To promote sustainable public health, the Finnish Food Authority recently recommended that adults consume 500–800 g of fruits, vegetables, and berries daily (https://urn.fi/URN:ISBN:978‐952‐343‐932‐0). A convenient source of fresh fruits and vegetables is ready‐to‐consume fresh‐cut produce, such as lettuce leaves. Fresh‐cut lettuce is typically washed, cut, packaged, and submitted to varied artificial temperature and light conditions during processing, transportation, and retail until purchase and consumption (Gil and Garrido 2020; Lareo et al. 2009; Peng and Simko 2023). Besides certain fruits that continue ripening during retail, most fresh fruits and vegetables begin to deteriorate and lose quality after harvest. This occurs rapidly with leafy vegetables such as lettuce due to the development of visual blemishes (i.e., cut‐surface discoloration), dehydration (i.e., tissue softening), senescence (i.e., leaf yellowing), and microbial spoilage (as reviewed by Peng and Simko 2023).

Solutions to extend fresh‐cut lettuce shelf life often involve cold temperatures in combination with modified atmosphere packaging (MAP), which creates a low oxygen (O_2_) and high carbon dioxide (CO_2_) environment (Peng and Simko 2023; Saltveit 2003). MAP extends vegetable storage by slowing respiration, reducing enzymatic leaf discoloration, delaying senescence, and inhibiting microbial growth (Chen et al. 2019; Guo et al. 2019; Ioannidis et al. 2018; Luna et al. 2016; Peng et al. 2020; Saltveit 2003). In MAP, quality deterioration of fresh‐cut lettuce still occurs involving dehydration and leaf yellowing as well as off‐odors (Haque et al. 2021; Ioannidis et al. 2018; Palumbo et al. 2024; Peng and Simko 2023; Tudela et al. 2013). However, these changes might not be easily detectable, especially within the first week of storage (Chen et al. 2019; Guo et al. 2019; Haque et al. 2021; Simko and Hayes 2018; Steensma et al. 2025; Tudela et al. 2013). Maintaining the desired gas composition in MAP requires balancing the product's physiological state with packaging permeability (Saltveit 2003). Furthermore, fluctuations in environmental parameters during storage such as light and temperature have been reported to impact the efficiency of MAP and therefore the quality of the product (Gil and Garrido 2020; Lareo et al. 2009; Martínez‐Sánchez et al. 2011; Peng et al. 2020; Saltveit 2003; Steensma et al. 2025). It has been demonstrated that when MAP is compromised by a defect in the package allowing for normal atmosphere (NA) or by light that stimulates photosynthetic O_2_ evolution inside the package, senescence resumes with chlorophyll degradation being one of the first signs (Chen et al. 2019; Gil and Garrido 2020; Martínez‐Sánchez et al. 2011; Steensma et al. 2025).

Though their use is still limited in postharvest research, chlorophyll *a* fluorescence (CF) analyses provide a powerful noninvasive tool to monitor physiology and quality of fresh‐cut products and thereby allow for better adapting storage conditions (Baldassarre et al. 2011; Herppich 2021). Several studies have employed CF methodology to assess the quality of green vegetables stored in MAP, including broccoli (DeEll and Toivonen 2003; DeEll and Toivonen 2000; Toivonen and DeEll 2001), spinach (Garrido et al. 2016), and lettuce (Hägele et al. 2016; Simko et al. 2015). Analogous studies have also been performed in apple fruit (Schlie et al. 2024; Wright et al. 2012; Wright et al. 2011). Most studies involving CF assays focused on determining quantum yield of Photosystem II (PSII) and nonphotochemical quenching (NPQ) and their correlation with postharvest quality (Garrido et al. 2016; Schlie et al. 2024). PSII quantum yield, particularly in dark‐adapted state (also known as Fv/Fm), has been widely used as a proxy for plant stress (Kalaji et al. 2014). NPQ is a protective mechanism that dissipates excessive absorbed light energy as heat, preventing damage to the photosynthetic apparatus. NPQ is sensitive to cellular ATP requirements and thus can be used for indirect estimate of plant energy metabolism (Bassi and Dall'Osto 2021).

Most, if not all, CF analyses of photosynthesis in MAP reported to date employed pulse amplitude modulated (PAM) fluorometry and the so‐called CF quenching assays where plants are exposed to various light intensities and dynamics of NPQ and PSII quantum yield are followed with saturating light pulses. Here we explored the applicability of another type of CF analyses, the flash‐induced chlorophyll fluorometry, to rapidly assess plant physiology directly inside packaging. In one of the methods called the OJIP test, polyphasic kinetics of CF rise during a saturating light pulse are measured with high time resolution. The O, J, I, and P inflections of this kinetics are sensitive to physiological processes in PSII (O–J), Photosystem I (I–P) or electron transfer processes between the two photosystems (J–I), making it a powerful tool to rapidly fingerprint the performance of the photosynthetic apparatus (Strasser et al. 2004).

Previously, we reported that continuous low light at 50 μmol m^−2^ s^−1^, in contrast to darkness, causes the headspace in MAP to revert toward atmospheric levels, thereby promoting senescence, although visual deterioration was minimal until after 7 days of storage (Steensma et al. 2025). Here we examined postharvest dynamics of photosynthesis in MAP during the first week of storage under conditions favorable (continuous darkness) and unfavorable (continuous light) to MAP, alongside perforated packaging allowing for NA as controls.

## 2 Materials and Methods

### 2.1 Plant Material

Nonheading crisp type lettuce ( *Lactuca sativa* ) var. “Crispyano” (Eazyleaf, Enza zaden, The Netherlands) was purchased from the farmhouse Robbes Lilla Trädgård Ab (Lindkoski, Finland). The lettuce plants were grown hydroponically for 2–3 weeks in a vertical farming system, whereafter they were replanted in the greenhouse maintained at 20°C and 75% relative humidity under an 18 h photoperiod and 6 h of darkness. If sun radiation fell under 200 W m^−2^, 150–190 mmol m^−2^ of supplemental LED lighting per day (70% red, 20% blue, 10% white) was used. Upon commercial maturity (40 days) the entire lettuce plants, including roots and substrate, were collected from the greenhouse the day before delivery, wrapped in protective plastic, and stored between 4°C and 10°C in darkness, and delivered the next day by cooled transportation (approximately 10°C). Upon arrival between 10 and 11 h 00 Eastern European Time (EET), the lettuce was kept at 4°C in darkness at delivery and taken out in batches followed by about 30 min at room temperature and ambient light during processing completed within 4 h after delivery.

### 2.2 Lettuce Processing, Storage, and Sample Collection

Lettuce leaf selection and processing was as described in Steensma et al. (2025). Briefly, leaves were carefully detached from the lettuce rosette, and two specific leaves in the middle position of the rosette were selected. The remaining stem tissue on the leaves (approximately 1 cm) was excised and discarded. The leaves were thereafter kept intact or cut perpendicularly to the midrib into 1 cm wide strips with a sharp knife, washed with cold tap water (Helsinki, Finland), spun with a manual salad spinner, followed by tap drying with absorbent tissue paper. The packaging film was donated by FreshServant Oy (Seinäjoki, Finland), a local distributor of fresh‐cut vegetable products in Finland. The film material was a complex of biaxially oriented polypropylene (20 μm) and antifog polypropylene (25 μm), with O_2_ and CO_2_ permeability less than 848 and 3389 cm^3^ m^−2^ 24 h^−1^, respectively. In the case of cut leaves, a half‐seam was added within the package to allow for the physical separation of midrib pieces from the rest of the leaf while maintaining the same headspace (Figure S1). Approximately 20 g of lettuce per 435 cm^2^ film was used, and the packages were sealed either under modified atmosphere conditions with an initial gas composition of 5% O_2_, 10% CO_2_, and 85% nitrogen (N_2_), using a Max F‐46 packaging machine (Vacuum Boss) or in perforated bags (six holes made with a syringe needle) to allow for NA (21% O_2_, 0.04% CO_2_, 78% N_2_) and sealed using a 520 ISTMED‐2 (Audion) impulse sealer. Fresh‐cut lettuce packages were either analyzed on the day of processing with no storage (0 day) or stored up to 7 days (7 days) in a climate chamber set at 7°C and 50% relative humidity, with one compartment maintaining darkness and another maintaining illumination with white LED. The temperature in both compartments was monitored using a HOBO Pendant MX Temperature/Light Data Logger and was found to be stable at 10°C under illumination, whereas 7°C was maintained in darkness. Two experimental sets (ES1 and ES2) were used in this study. Light intensity ranged between 30 and 53 μmol m^−2^ s^−1^ for ES1 and 40–54 μmol m^−2^ s^−1^ for ES2. To avoid positional effects under illuminated conditions, the samples were randomly redistributed on the shelves at least once on every data collection day.

ES1 (June 2023) was used for headspace determination, weight loss, chlorophyll determination by hyperspectral imaging (HSI), and chlorophyll fluorescence analyses of both intact and cut leaves. Lettuce plants were delivered and processed on three consecutive days constituting three experimental replicates with one biological replicate per storage condition and day of storage analyzed.

ES2 (October 2024) was used for headspace determination and thylakoid isolation of intact leaves. All lettuces were delivered and processed on the same day, with four biological replicates per storage condition and day of storage analyzed.

### 2.3 Chlorophyll Fluorescence Analyses

All CF analysis were performed on ES1 in a dark room. For the cut lettuce, the leaf area was standardized by separating midrib pieces from the rest of the leaf within the same package and only the nonmidrib pieces were analyzed. Additionally, intact leaves were analyzed to focus on the central leaf area. Flash‐induced (OJIP) chlorophyll fluorometry was performed after approximately 45 min of dark adaptation in room temperature, through the packaging in the center of the abaxial side of intact lettuce leaves or nonmidrib pieces of cut leaves (Figure S1B) in a dark room using a FluorPen FR 100 fluorometer (Photon Systems Instruments). The measurement routines were performed according to the manufacturer's instructions. The selected CF parameters were recalculated based on the corrected J‐inflection timing as described in section “First and second‐order derivatives of the OJIP and Vt transient” (Table S1). All samples were analyzed in a span of less than 10 min starting around 14 h 00 (EET) each day of analysis. The same samples were further stored in darkness for a minimum of 10 min for the first sample to a maximum of 4 h 40 min for the last sample before being analyzed by PAM CF imaging using IMAGING‐PAM M‐series (Walz). After dark acclimation, minimal (Fo) and maximal (Fm) fluorescence was determined. Fm was allowed to relax for 1 min, after which low‐intensity light (ca. 40 μmol m^−2^ s^−1^) was turned on for 4 min. Steady‐state fluorescence (Fs) was followed over this period with measuring light pulses. Next, a saturating light pulse was triggered to address light‐adapted maximal fluorescence (Fm′). After the low‐light phase, actinic light intensity was increased to ca. 450 μmol m^−2^ s^−1^ for 3 min, with saturating flashes programmed once a minute to measure Fm′. After the high light period, relaxation of Fm′ was followed in darkness for 3 min. NPQ was calculated as: NPQ = (Fm − Fm′)/Fm′ (Horton and Ruban 1992).

### 2.4 Determination of Chlorophyll Content

Chlorophyll content was determined from ES1 under ambient light (after CF analysis steps) as described in Steensma et al. (2025) by HSI with Specim IQ camera (0604675, SPECIM Spectral Imaging Oy Ltd.) and Vogelmann 3 (Vog3) hyperspectral index on reflectance data of each sample (Vogelmann et al. 1993). The index is based on red edge location in the reflectance spectrum and calculated as a division of two hyperspectral reflectance bands, 715 and 705 nm: Vog3 = R715/R705. The sharp rise of leaf spectra in the red‐light wavelength region, and its precise location is shown to be a reliable predictors of chlorophyll content and therefore Vog3 is one of the best estimators for total chlorophyll content (Fillela and Penuelas 1994; Main et al. 2011).

### 2.5 First and Second‐Order Derivatives of the OJIP and Vt Transient

To obtain time‐adjusted OJIP parameters, we determined first and second‐order derivatives of the OJIP and Vt transient as described in (Akinyemi et al. 2023) with modifications. We used the Savitzky–Golay method for smoothing and calculating the first, second, and third derivatives (Press et al. 2007; Savitzky and Golay 1964). We iterated the smoothing algorithm over the smoothing rate until achieving (*n* < 6) zero points for the third derivative and (*n*–1) zero points for the second derivative in the range (min [third derivative zero points]–max [third derivative zero points]). The plateaus and rises were defined as follows. If the second derivative was negative and the third derivative was zero, this was the starting point of a plateau. If the second derivative was positive and the third derivative was zero, this was the starting point of a rise. By enforcing the (*n*–1) second derivative zero points, we ensured that the second derivative was of different signs at two consecutive third derivative zero points and, thus, achieved alternation of plateaus and rises. The CF parameters recalculated based on the corrected J‐inflection timing are shown in Table S1.

### 2.6 Determination of Headspace and Weight Loss

In ES1, headspace gases (O_2_ and CO_2_) were determined (after CF and HSI analyses) with a CheckMate 9900 O_2_/CO_2_ gas analyzer (PBI‐Dansensor). In ES2, headspace gases were determined prior to thylakoid isolation with a GS3W zirconia detector gas analyzer from Sensorcell (Systech Illinois). Due to instrument error, the detection limit for CO_2_ gas was 1% for ES2. Weight loss was determined at ES1 with a scale (Precisa 1000 C–3000 D, Precisa Gravimetrics AG, Dietikon) with the leaf material outside the package. The weight was measured twice, first at 0 day and again at subsequent storage time points.

### 2.7 Thylakoid Isolation and Blue Native Gel

Samples from ES2 were kept in darkness at room temperature for 1 h before thylakoid extraction, which started between 12 h 00 and 15 h 00 (EET) each day of analysis. Thereafter, leaves were ground in ice‐cold grinding buffer (50 mM Hepes‐NaOH pH 7.5, 330 mM sorbitol, 5 mM MgCl_2_, 0.05% (w/v) BSA and 10 mM NaF) and filtered through Miracloth (Millipore). Chloroplasts were collected by centrifugation at 1350 g for 5 min at 4°C and osmotically ruptured in ice‐cold shock buffer (50 mM Hepes‐NaOH pH 7.5, 5 mM sorbitol, 10 mM MgCl_2_ and 10 mM NaF). The released thylakoid membranes were collected by centrifugation at 1350 g for 5 min at 4°C, washed twice with storage buffer (50 mM Hepes‐NaOH pH 7.5, 100 mM sorbitol, 10 mM MgCl_2_ and 10 mM NaF), and finally resuspended in storage buffer.

Isolated thylakoids (60 μg protein) were suspended in ice‐cold 25BTH20G buffer (25 mM BisTris‐HCl pH 7.0, 20% (v/v) glycerol and 0.25 mg ml^−1^ Pefabloc). Resuspended thylakoids were solubilized with an equal volume of 2% β D‐dodecyl maltoside (β‐DM) in 25BTH20G buffer for 5 min on ice. Insoluble material was removed by centrifugation at 16,000 g for 20 min at 4°C, and 1/10 of the volume of loading buffer (100 mM BisTris‐HCl pH 7.0, 0.5 M aminocaproic acid, 30% [w/v] sucrose and 50 mg ml^−1^ Serva Blue G) was added to the supernatant. Solubilized thylakoid protein complexes were separated on 3%–12% acrylamide gradient gels according to Järvi et al. (2011). Separated pigment‐protein complexes were imaged using an office scanner.

### 2.8 Western Blotting

Thylakoid membrane proteins were separated by SDS‐PAGE (15% acrylamide) and transferred to a polyvinylidene difluoride membrane (Immobilon). The membranes were then blocked with bovine serum albumin or milk, incubated with rabbit antiphosphothreonine antibodies (New England Biolabs) or anti‐PsbS (Agrisera). The blots were incubated with horseradish peroxidase‐conjugated secondary antibody, and the signal was visualized by ECL Prime chemiluminescence reagents (Cytiva).

### 2.9 Data Rendering and Analysis

Data rendering and statistical analysis was performed using Origin (Pro), Version 2024 (OriginLab Corporation), Progenesis QI 2.3 software (Umetrics) as well as Microsoft PowerPoint and Microsoft Excel 2021 Version 2402 (Microsoft corporation).

## 3 Results

### 3.1 Fv/Fm Does Not Reflect Deterioration of Fresh‐Cut Lettuce Under Modified Atmosphere Packaging

Previously, we established that exposing fresh‐cut lettuce in MAP to constant low light reverted the headspace gas composition toward NA and activated a transcriptional response with hallmarks of leaf senescence within the first week of storage, despite minor visual symptoms of deterioration (Steensma et al. 2025). In contrast, dark storage preserved MAP and delayed senescence. The light‐induced changes suggested a role for photosynthesis in the observed physiological responses.

**FIGURE 1:**
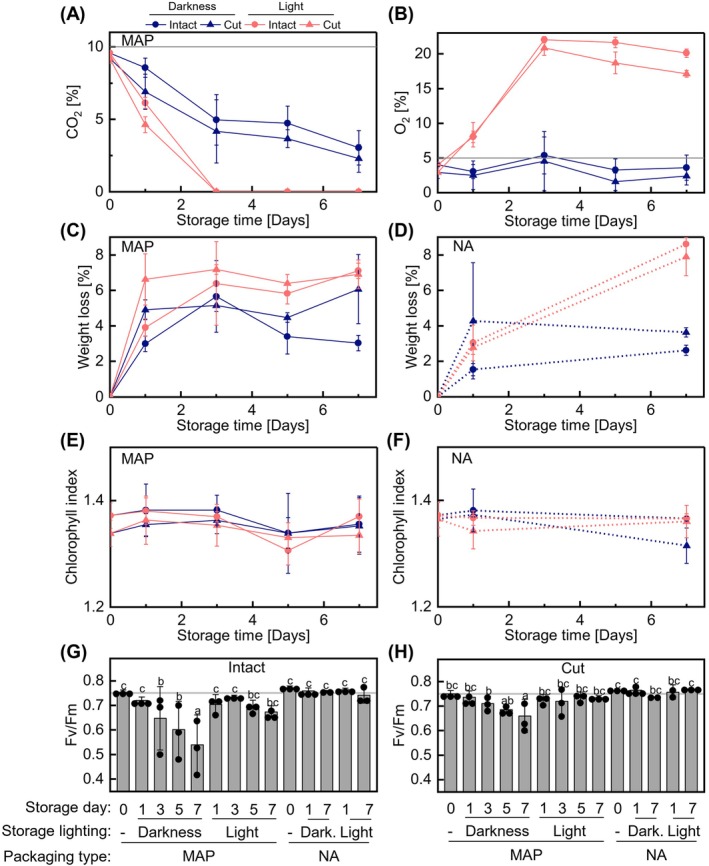
Postharvest changes in fresh‐cut lettuce during storage in MAP or NA. Changes in headspace CO_2_ (A) and O_2_ (B) levels in MAP containing intact or cut detached lettuce leaves just after packaging on day 0 (0 day) with no storage and up to 7 days of storage under darkness at 7°C or under 30–52 μmol photons m^−2^ s^−1^ light at 10°C (7°C initial setting) and 50% RH. The initial MAP conditions were set to 5% O_2_ and 10% CO_2_ as indicated by the grey line. Changes in weight in MAP (C) and NA (D); in leaf chlorophyll index in MAP (E) and NA (F) of samples described in (A) as well as similar samples packed in NA (21% O_2_ and 0.04% CO_2_). Fv/Fm determined by measuring flash‐induced CF (OJIP) in intact (G) and cut (H) leaf samples described in (A–F). Statistical analysis was performed using a two‐way ANOVA followed by Fisher LSD testing (different letters indicate *p* ≤ 0.05) and the grey line indicates the mean value for 0 day in MAP. The data (ES1) represents the mean ± SD across two to three time‐independent experimental replicates with one biological replicate each. Due to experimental purposes the samples spent 4–7 h in darkness at room temperature before measurement of parameters shown in A–F.

We thus employed spectroscopic methods to assess early‐stage photosynthetic changes in MAP or NA in darkness or light. Concurrently, headspace O_2_ and CO_2_ levels were monitored. Cut or intact leaves were stored in MAP and analyzed at days (d) 0 day, 1 day, 3 days, 5 days, and 7 days (for MAP), or at 0 day, 1 day, and 7 days (for NA control). In line with our previous study (Steensma et al. 2025), both experimental sets used in this study showed initial MAP levels of 10% CO_2_ and 5% O_2_ in the packaging headspace, which reverted toward atmospheric levels by 7 days of storage under light. In contrast, under dark storage, MAP conditions were better maintained, although gas concentrations still declined to approximately 2%–3% CO_2_ and 2%–4% O_2_ in packages containing intact or cut leaves. The reduction in CO_2_ is likely due to higher permeability of the packaging to CO_2_ compared to the rate of its production through respiration (Figure 1A). Notably, at 1 day, O_2_ and CO_2_ levels were around 8% and 5%–6%, respectively, in both kinds of packages (Figure 1B). Weight loss of the leaf material was slightly enhanced under light compared to darkness in packages originally sealed with MAP, while more pronounced differences were observed at 7 days in NA (Figure 1C,D). However, no significant changes in chlorophyll contents were detected (Figure 1E,F), in line with minimal deterioration.

To follow the changes in photosynthesis in situ, we first assayed the maximal quantum yield of PSII (Fv/Fm), since this parameter is widely used in the literature as the indicator of plant stress. Intriguingly, only in MAP in darkness, where quality is known to be best preserved, did we find a clear decrease in Fv/Fm over time with 1.4‐fold and 1.1‐fold decreases at 7 days compared to 0 day in intact and cut leaves respectively (Figure 1G,H). This indicated that Fv/Fm was not a reliable metric to assess packaged fresh‐cut lettuce in terms of typical quality losses. The changes were similar in intact and cut leaves, thus we report only intact leaf CF results hereafter.

### 3.2 Dark‐Storage and MAP Cause Drastic Changes in Chlorophyll Fluorescence

**FIGURE 2:**
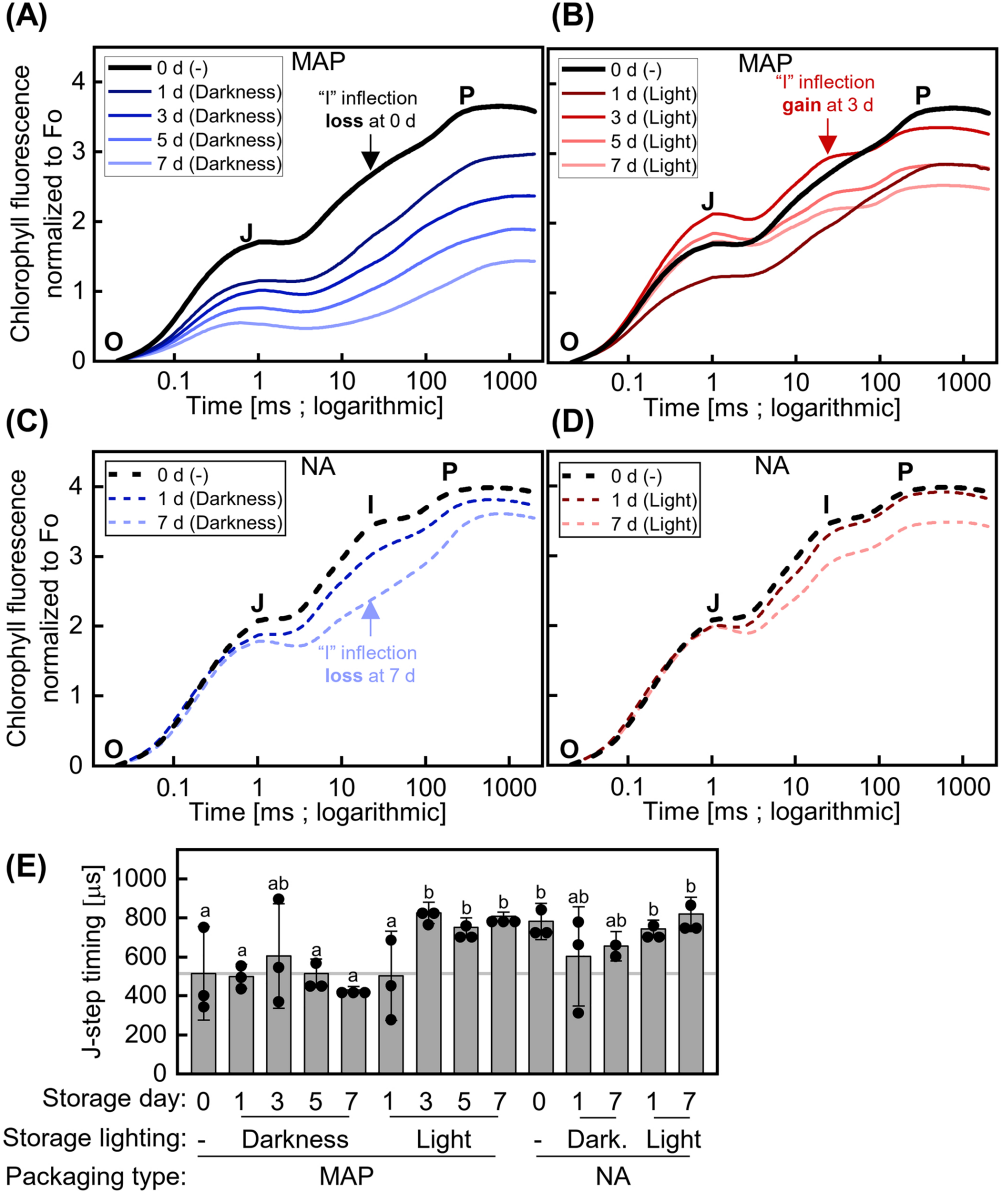
Flash‐induced CF (OJIP) kinetics of fresh‐cut lettuce during storage in MAP or NA. (A–D) Normalized OJIP kinetics of intact detached lettuce leaves packed in MAP (A, B) or NA (C, D) just after packaging on day 0 (0 day) with no storage (−) and up to 7 days of storage under darkness at 7°C (A, C) or 30–52 μmol photons m^−2^ s^−1^ light (L) at 10°C (7°C initial setting) (B, D) and 50% RH. Key “I” inflection events are indicated with arrows. (E) Timing of the J inflection of kinetics shown in A–D. Statistical analysis was performed using a two‐way ANOVA followed by Fisher LSD testing (different letters indicate *p* ≤ 0.05) and the grey line indicates the mean value for 0 day in MAP. The data (ES1) represent the mean (A–D) or mean ± SD (E) across two to three time‐independent experimental replicates with one biological replicate each. Throughout storage, average gas compositions were 21% O_2_ and 0.04% CO_2_ in NA and varied from the initial 5% O_2_ and 10% CO_2_ in MAP as shown in Figure 1A,B. By 7 days in MAP, gas compositions were 3.6% ± 1.8% O_2_ and 3.0% ± 1.2% CO_2_ in darkness and 20.1% ± 0.6% O_2_ and 0.0% ± 0.0% CO_2_ in light.

To explore the nature of the changes in Fv/Fm (Figure 1), we analyzed the polyphasic kinetics of flash‐induced CF rise known as the OJIP test that was recorded while determining Fv/Fm. The results revealed the changes in photosynthesis at different time scales. In line with lowering Fv/Fm (Figure 1G,H), the intensity of normalized CF signal progressively decreased from 0 to 7 days in MAP in darkness, which did not happen in light (Figure 2A,B). Interestingly, the changes were similar between MAP dark and light‐stored leaves on 0 and 1 days (Figure 2A,B), when the O_2_ and CO_2_ levels were still different from NA (Figure 1A,B). However, from 3 to 7 days, normalized CF intensity continued to decline in MAP in darkness, whereas in MAP in light it increased from 1 to 3 days and then slightly decreased from 3 to 7 days (Figure 2A,B).

The described decrease in overall CF intensity did not happen in NA; however, the “I” inflection gradually decreased under prolonged dark storage, but not light storage (Figure 2C,D). In striking contrast with NA, in MAP the “I” inflection disappeared already 1 h after packaging with no storage (0 day). The loss of “I” inflection was observed at 1 day for leaves stored in MAP both in darkness and light, but it reappeared by 3 days under light storage (Figure 2A,B). These changes were paralleled with the altered timing of the “J” inflection measured after the start of the light flash. The “J” inflection occurred significantly sooner in dark‐stored samples in MAP at all days including 0 day, but only at 1 day in light‐stored samples in MAP (Figure 2E). Overall, the above results revealed: (1) long‐term decrease in normalized CF intensity in MAP in darkness (1–7 days) and in light (1 day only); (2) slow disappearance of the “I” inflection in NA in darkness; and (3) rapid multiple changes in OJIP kinetics including the accelerated “J” and the disappearing “I” inflections that were triggered by MAP and persisted until 7 days in darkness, but restored by light storage in MAP after 1 day.

**FIGURE 3:**
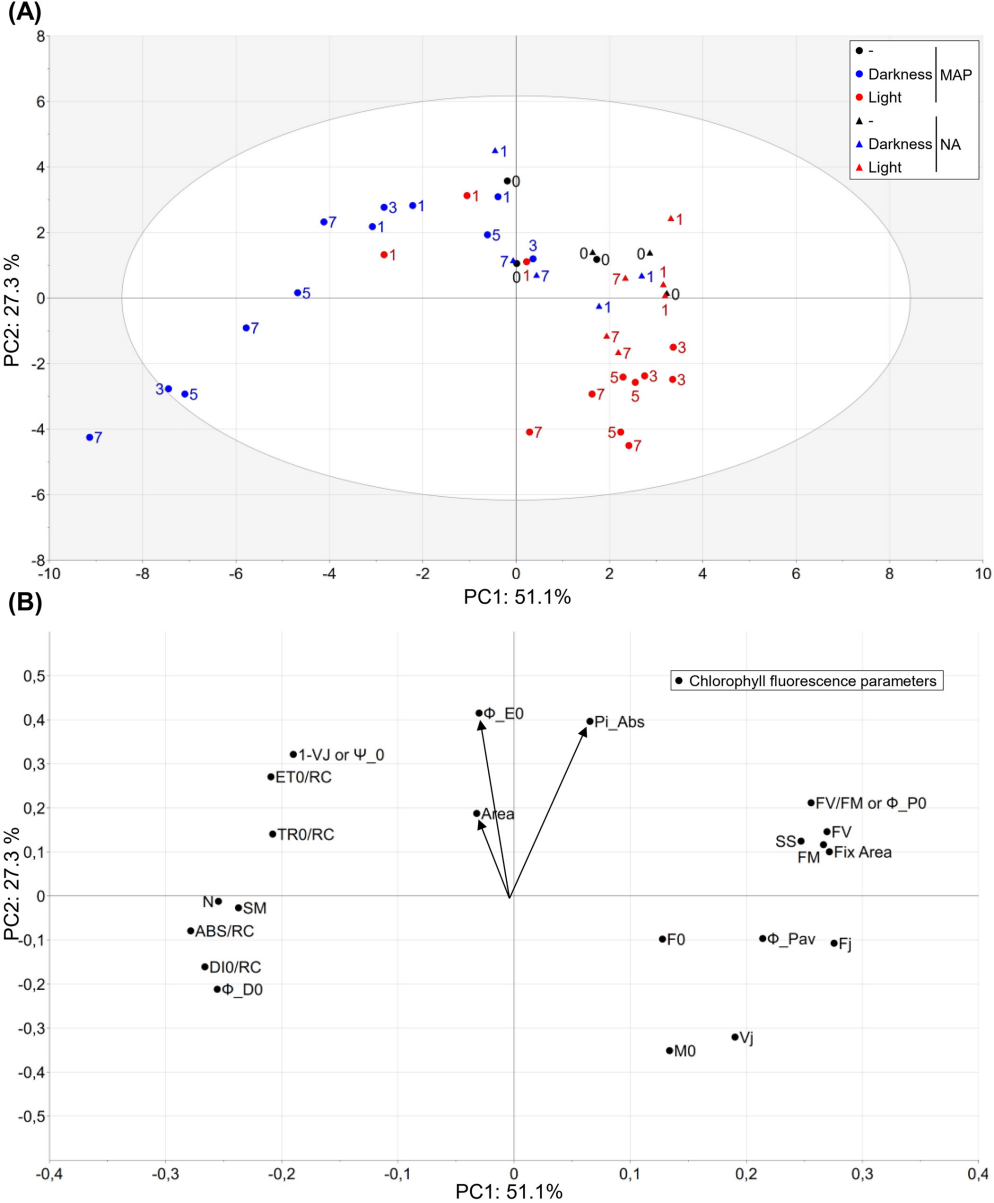
Photosynthetic parameters derived from OJIP kinetics discriminate storage time and storage condition in fresh‐cut lettuce. (A) PCA score plot describing the differences between intact detached lettuce leaves packed in NA (triangles) or MAP (circles) just after packaging on day 0 (0 day) with no storage (−, black symbols) and up to 7 days of storage under darkness (blue symbols) at 7°C or 30–52 μmol photons m^−2^ s^−1^ light (red symbols) at 10°C (7°C initial setting) and 50% RH. (B) Photosynthetic parameters calculated from OJIP kinetics and influencing the corresponding principal components shown in (A). The data (ES1) represents two to three time‐independent experimental replicates with one biological replicate each. Throughout storage, average gas compositions were 21% O_2_ and 0.04% CO_2_ in NA and varied from the initial 5% O_2_ and 10% CO_2_ in MAP as shown in Figure 1A,B. By 7 day in MAP, gas compositions were 3.6% ± 1.8% O_2_ and 3.0% ± 1.2% CO_2_ in darkness and 20.1% ± 0.6% O_2_ and 0.0% ± 0.0% CO_2_ in light.

OJIP CF kinetics can be used to calculate many parameters that have been linked to various photosynthetic functions (Strasser et al. 2004; Figure 3, Table S1, Figure S2). We subjected these parameters to principal component analysis (PCA). PC1 and PC2 explained 51.1% and 27.3% of the variation, respectively (Figure 3A). PC1 represented progressive changes occurring in MAP in darkness and largely prevented by light storage. PC2 reflected more subtle changes that occurred under both dark‐ and light‐adapted storage. Only three parameters, Area, Performance index (PI_ABS_), and Ф_E0, appeared to best contribute to PC2. PI_ABS_ showed a marked decrease with time in MAP in light storage; a similar trend was observed under other conditions with no statistical significance (Figure 3B, Figure S2). We conclude that other parameters than Fv/Fm, such as PI_ABS_, may be better markers of postharvest deterioration of lettuce.

### 3.3 MAP Leads to Elevated Steady‐State CF in Light‐Adapted State and Compromised CF Relaxation

**FIGURE 4:**
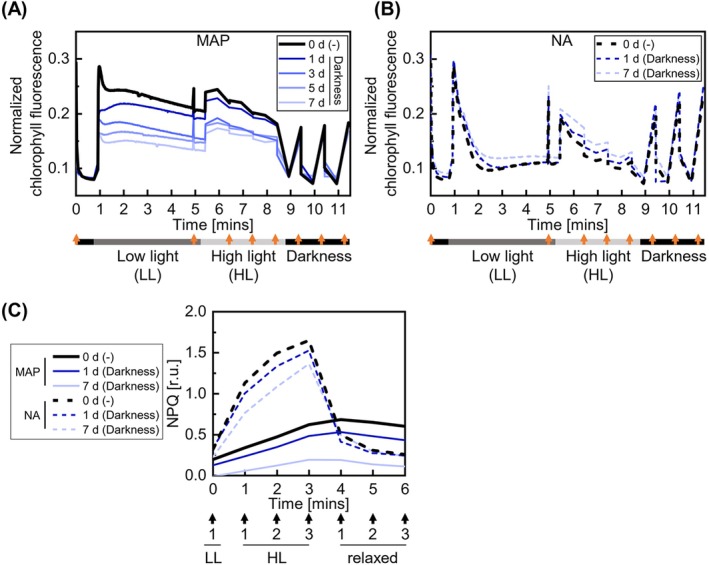
PAM CF analysis in fresh‐cut lettuce during storage under darkness in MAP or NA (A, B, accordingly). Light/dark conditions (gray/black boxes) and saturating light pulses (orange arrows) are indicated below the plot. (C) The corresponding NPQ dynamics in lettuce leaves packed in NA or MAP just after packaging on day 0 (0 day) with no storage (−) and up to 7 days of storage under darkness at 7°C and 50% RH. The data (ES1) represent the mean across two to three time‐independent experimental replicates with one biological replicate each. Throughout storage, average gas compositions were 21% O_2_ and 0.04% CO_2_ in NA and varied from the initial 5% O_2_ and 10% CO_2_ in MAP as shown in Figure 1A,B. By 7 day in MAP, gas compositions were 3.6% ± 1.8% O_2_ and 3.0% ± 1.2% CO_2_ in darkness.

OJIP analyses revealed fast operational effects of MAP on the photosynthetic apparatus likely linked to the composition of the leaf gas environment. Furthermore, prolonged storage in darkness in MAP led to further major structural changes in photosynthesis. To gain mechanistic understanding of these effects, we performed in vivo quenching assays using PAM CF imaging. Dark‐acclimated leaves inside the packages were exposed to 4 min of low‐intensity light, followed by 3 min of high‐intensity light and 3 min of darkness. The dynamics of CF yield were recorded with measuring light pulses (Figure 4A,B); NPQ was addressed with saturating light flashes triggered once a minute (Figure 4C).

Exposure to low‐intensity light led to the induction and concomitant relaxation of CF referred to as Kautsky kinetics. Kautsky kinetics is sensitive to many photosynthetic processes, including light harvesting, photosynthetic electron flows, and carbon assimilation reactions (Stirbet et al. 2014; Stirbet and Govindjee 2011; Tyystjärvi et al. 1999). After 4 min of exposure to low light, CF approached its steady‐state level Fs (time 5 min in Figure 4A,B). In leaves exposed to MAP, Fs was dramatically increased as early as 0 day, whereas in leaves stored in NA in darkness, Fs remained comparatively low and unaffected by the storage duration (Figure 4A,B). On 1–7 days of dark storage in MAP, Fs progressively decreased but remained higher than in NA. Interestingly, in samples stored in MAP under light, the same effect was only observed on 0 day and to a lesser extent on 1 day while CF kinetics became indistinguishable from that in NA thereafter (Figure S3A,B). These results agreed with the operational effects of MAP on leaf photosynthesis as assessed with OJIP (Figure 2). Elevated Fs likely reflected compromised electron sinks for photosynthetic electron flows, which disabled the relaxation of CF. The progressive decrease in Fs levels during dark storage 0–7 days in MAP was most likely associated with gradual suppression of light harvesting and photochemical reactions.

MAP atmosphere also strongly affected the dynamics of NPQ (Figure 4C, Figure S3C,D). Exposure to high light led to lower NPQ in MAP than in NA. This effect of MAP was visible already on 0 day and worsened during days 1–7 days of dark storage (Figure 4C). Under light storage, NPQ reverted to values similar to those in NA (Figure S3C,D). Fast changes in NPQ assessed in this experiment, the so‐called energy‐dependent component of NPQ (qE), largely depend on the proton gradient (ΔpH) across chloroplast thylakoid membranes. ΔpH is linked to the activity of the chloroplast ATP synthase: the more active this enzyme is, the more it contributes to the dissipation of ΔpH and thereby the decrease in qE. Thus, the observed changes in NPQ may indicate higher activity of the chloroplast ATP synthase in MAP storage conditions. We hypothesize that this reflects altered cellular energy metabolism, probably caused by impaired respiration under O_2_‐limited atmosphere. Long‐term decay of NPQ during dark storage in MAP coincided with the changes in OJIPs (Figure 2) and Fs (Figure 4A). It was likely due to the progressive inhibition of photochemical reactions of yet unclear nature.

### 3.4 Photosynthetic Thylakoid Protein Composition Cannot Explain the Functional Changes in MAP Storage

**FIGURE 5:**
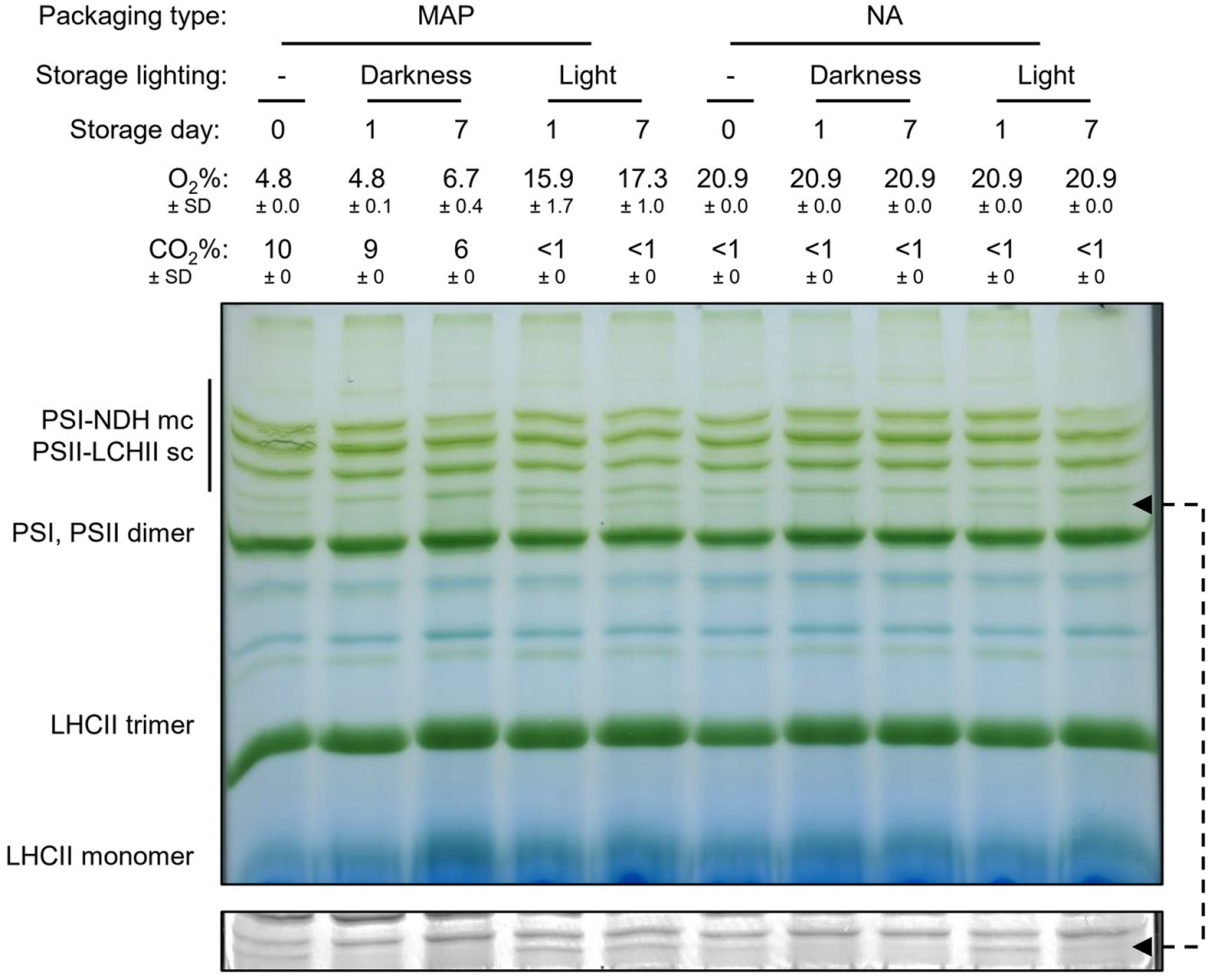
Changes in the composition of photosynthetic complexes during storage in NA or MAP. Representative blue‐native gel electrophoresis of solubilized thylakoids that were isolated from intact detached lettuce leaves (ES2) just after packaging on day 0 (0 day) with no storage (−) and up to 7 days of darkness at 7°C or 40–54 μmol photons m^−2^ s^−1^ light at 10°C (7°C initial setting) and 50% RH. The O_2_ and CO_2_ data represent the mean ± SD across three to four biological replicates. Bottom panel: A contrasted fragment of the image revealing a putative PSII‐LHCII antenna complex characteristic for dark storage.

We examined whether the observed changes in CF parameters were associated with alterations in the abundance of photosynthetic pigment–protein complexes. The BN‐PAGE separation displayed a pattern of thylakoid protein complexes comparable to the pattern typically observed for the model plant *Arabidopsis thaliana* (Figure 5; Järvi et al. 2011). In the samples loaded based on equal protein amounts in each thylakoid preparation, the relative abundance of the protein complexes remained stable across storage conditions. The only detected exception was the band that likely corresponded to a complex of PSII with its light‐harvesting antennae (LHCII) marked by an arrow in Figure 5 and shown under higher contrast in the bottom panel of the same figure. This complex was reproducibly detected under dark storage conditions both in NA and MAP. We hypothesize that it can be functionally linked to the CF changes observed in OJIP assays (Figures 2 and 3, Figure S2). Importantly, the results did not reveal any major protein–pigment complexes characteristic of MAP. This indicated that the fast operational effects of MAP on photosynthesis were not caused by any large‐scale rearrangements of photosynthetic complexes.

We next denatured thylakoid samples, separated protein in SDS‐PAGE, and stained with the antiphosphothreonine antibodies (αP‐Thr) detecting phosphorylation levels of the photosynthetic complexes (Figure S4). Phosphorylation of PSII subunits D1, D2, and PsbH is implicated in their maintenance and repair cycle (Riché et al. 2019; Vener 2007), while phosphorylation of LHCII is sensitive to many physiological parameters, including the redox state of the photosynthetic electron transfer chain (more specifically, the plastoquinone pool) and relative excitation of Photosystems I and II (Longoni and Goldschmidt‐Clermont 2021). We observed increased phosphorylation of both PSII core and LHCII subunits in light storage conditions in both NA and MAP. Dark storage under both atmospheres led to gradual dephosphorylation of LHCII, most probably indicating a lower reduction state of the plastoquinone pool. Unexpectedly, we did not detect any changes specific to the NA or MAP atmosphere. Finally, we addressed the abundance of the thylakoid protein PsbS that is responsible for the establishment of the qE component of NPQ. No difference in PsbS abundance was detected between the treatments (Figure S4). To summarize, the functional alterations observed in photosynthetic electron transfer in Figures 1, 2, 3, 4 could not be attributed to any major structural changes in the photosynthetic protein complexes.

## 4 Discussion

Chlorophyll fluorometry provides a powerful noninvasive approach for assessing the physiological status of packaged fresh produce. In this study, we applied this method to phenotype fresh‐cut lettuce in MAP during 1 week of storage under both favorable (continuous darkness) and unfavorable (continuous light) conditions, where visual deterioration was minimal or absent (Figure 1). Our results revealed distinct physiological responses of various CF parameters to MAP and normoxic storage conditions. Surprisingly, MAP rapidly altered photosynthetic light reactions, triggering multiple changes in OJIP kinetics (Figures 2 and 3), decreasing NPQ and increasing Fs (Figure 4). The loss of MAP under light storage coincided with the reversion of these parameters to levels characteristic of normal conditions. Additional changes occurred during prolonged dark storage in MAP, including a progressive decline in CF signal intensity (Figure 2), a further reduction in NPQ, and a decrease in Fs (Figure 4). We hypothesize that these shifts are linked to the gradual suppression of light harvesting and photochemical reactions, the precise nature of which remains unclear. Whether the observed photosynthetic adjustments arise from alterations in chloroplast redox metabolism or the functional status of the ATP synthase remains to be established. In contrast, long‐term dark storage in NA resulted in less pronounced changes in OJIP kinetics (Figures 2 and 3), with the most notable effect being the suppression of the “I” inflection. Overall, these results emphasize the potential of CF as a tool for optimizing storage conditions and extending the shelf life of fresh‐cut produce.

Interestingly, the OJIP‐derived parameters Area over the OJIP kinetics, Performance index (PI_ABS_), and quantum yield of electron transport (Ф_E0) emerged as more reliable markers of postharvest deterioration of lettuce than the widely used Fv/Fm. PI_ABS_ has previously been shown to be a more sensitive indicator of plant stress than Fv/Fm (Živčák et al. 2014). Our results highlight the need for caution when interpreting Fv/Fm in studies of photosynthesis in MAP. This parameter may overlook crucial details of the Fo‐Fm chlorophyll fluorescence rise, as indicated by the OJIP kinetics in MAP versus NA (Figure 2) and bias the prediction related to lettuce quality. Recent advances have revisited underlying mechanisms of the OJIP kinetics, for example, by the discovery of dielectric processes within PSII (Sipka et al. 2021), the possible implication of Photosystem I fluorescence during the I‐P rise (Schreiber and Klughammer 2021) and the wavelength dependence of Fv/Fm (Nanda et al. 2024). Thus, further research is needed to establish a mechanistic interpretation of the OJIP kinetics.

The shifts in CF parameters under transitions between MAP and normoxic conditions indicated that photosynthesis was highly sensitive to changes in the gaseous environment. Although this study focused on a single lettuce cultivar, our findings align with previous studies on broccoli (Toivonen and DeEll 2001) and apple (Schlie et al. 2024) suggesting that similar results may be reproducible in other lettuce cultivars and leafy vegetables. Furthermore, this observation is important to consider in further studies of plant physiology in MAP, as a number of the previously reported analyses were carried out in plant material taken out of the packaging. MAP atmosphere provides plants with a highly unnatural gas environment, which can directly impact plant energy metabolism, including photosynthesis, respiration, photorespiration, and fermentation; affect plant senescence, synthesis of secondary metabolites, and resistance to biotic stress. For example, low cellular levels of O_2_ can suppress mitochondrial respiration (Igamberdiev and Hill 2009). This causes activation of fermentative metabolism (Tudela et al. 2013), which may supply reducing equivalents to the photosynthetic electron transfer chain, namely the plastoquinone pool (Nellaepalli et al. 2012). Reduction of the plastoquinone pool has been linked to NPQ and phosphorylation of LHCII (Havaux 2020). In this study, we observed neither enhanced NPQ nor increased LHCII phosphorylation in MAP. One possible reason for this is the relatively short storage time, which did not allow for the development of fermentative metabolism. Nevertheless, low‐O_2_ metabolism appears to be prevalent in fresh‐cut lettuce stored under darkness in MAP already within the first week of storage (Steensma et al. 2025). Therefore, a role for fermentative pathways at later storage stages cannot be excluded. This represents an interesting avenue for further investigation, particularly since, despite the overall preservation of lettuce quality in terms of color and weight (Figure 1), off‐odor development may still impact consumer acceptability (Tudela et al. 2013). Furthermore, the sensitivity of CF to shifts from a modified to normoxic atmosphere could serve as a promising tool for detecting compromised MAP packaging at the retail level. Nonphysiologically high levels of CO_2_ also affect multiple cellular functions, including respiration (Bhargava and Mitra 2021), photorespiration (Eisenhut et al. 2019), and photosynthesis. For example, CO_2_ plays a crucial role in modulating electron transfer reactions directly within PSII (Fantuzzi et al. 2022; Shevela et al. 2012). At the electron acceptor side of PSII, it can alter the redox properties of the quinone electron acceptors Q_A_ and Q_B_, impacting overall charge recombination kinetics. On the electron donor side of PSII, it may influence water oxidation and the stabilization of the O_2_‐evolving complex, although the latter role remains disputed (Shevela et al. 2012). Thus, CO_2_ actively participates in regulating PSII functions and maintaining efficient energy conversion. It should be noted that CO_2_ and O_2_ levels evolved during dark storage under MAP, making it difficult to fully separate their effects on CF from those of storage duration. Future studies using controlled atmosphere conditions could help disentangle these variables and clarify the specific impact of gas composition on PSII function.

To our surprise, we did not see any changes in the composition of photosynthetic complexes that could explain the dramatic changes in CF in MAP. The physiological reasons for these reactions remain to be determined, but our results suggest that they may be associated with the above‐described direct effects of O_2_ or CO_2_ on photosynthetic apparatus. Overall, a molecular‐level understanding of the effects of headspace gas composition on plant energy metabolism is necessary to develop new methods of postharvest storage and quality control.

## Author Contributions

The conceptualization and development of methodologies were carried out by P.S., A.S., J.P.V., and L.A. The software, VF design, and investigation were performed by P.S., A.S., J.P.V., and L.A. Validation, formal analysis, data curation, visualization, and writing of the original draft were conducted by P.S., A.S., J.P.V., and S.K. Writing, including review and editing, and supervision, were undertaken by P.S., A.S., S.K., and K.S.M. Project administration was managed by K.S.M. Resources were provided by A.S., S.K., and K.S.M.; funding by A.S., S.K., and K.S.M.

## Supporting information


**Data S1:** ppl70433‐sup‐0001‐Supinfo.pdf.

## Data Availability

Data that supports the findings of this study are available in Supporting Information of this article.
