# Supplementary material for: Postharvest Dynamics of Photosynthesis in Fresh‐Cut Lettuce
Source: Physiol Plant. 2025 Aug 3;177(4):e70433. doi: 10.1111/ppl.70433 (PMC12319283; doi:10.1111/ppl.70433)
Supplement: Supplementary file 1 — Data S1: ppl70433‐sup‐0001‐Supinfo.pdf. [file PPL-177-e70433-s001.pdf]

F_0	Minimal fluorescence intensity from a dark-adapted leaf
F_j	Fluorescence intensity at J-step
F_M	Maximal fluorescence intensity
F_V	Maximal variable fluorescence from a dark-adapted leaf: $F_M - F_0$
V_j	Relative variable fluorescence intensity at J-step: $(F_J - F_0) / (F_M - F_0)$
M_0	Approximated initial slope of the fluorescence transient: $TR_0/RC - ET_0/RC = 4 (F_{300} - F_0) / F_M - F_0)$
Area	Area between fluorescence curve and Fm (background subtracted)
Fix Area	Area below the fluorescence curve between $F_{40\mu s}$ and F_{1s} (background subtracted)
S_M	Area / ($F_M - F_0$) (multiple turn-over)
S_S	the smallest S_M turn-over (single turn-over)
N	turn-over number Q_A: S_M . M_0 . (1 / V_J)
F_V/F_M or Φ_P_0	Trapping probability or maximum quantum yield of primary photochemistry of a dark-adapted leaf. This shows the probability that the Photosystem II reaction centre will trap an absorbed photon. Fv/Fm is valuable for monitoring the functioning of the Photosystem II.
$1-V_J$ or Ψ_0	Probability with which a PSII trapped electron is transferred beyond QA
Φ_E_0	$(1 - (F_0 / F_M)) . \Psi\_0$
Φ_D_0	$1 - \Phi\_P_0 = (F_0 / F_M)$
Φ_Pav	$\Phi\_P_0 (S_M / t_{Fm})$ t_{Fm} = time to reach Fm (in ms)
Pi_Abs	Performance Index (potential) for energy conservation from photons absorbed by Photosystem II to reducing intersystem electron acceptors: RC/ABS [$F_V/F_M / (1- F_V/F_M)$] [1- V_J / 1- (1-V_J)]
ABS/RC	Apparent antenna size of an active PSII: M_0 . (1/ V_J) . (1/Φ_P_0)
TR_0/RC	Trapping flux leading to QA reduction per reaction centre: M_0 . (1/ V_J)
ET_0/RC	Electron transport in an active reaction centre: M_0 . (1/V_J) . Ψ_0
DI_0/RC	Dissipation of energy per reaction center: (ABS / RC) – (TR_0 / RC)

SUPPLEMENTAL TABLE 1. Chlorophyll fluorescence parameters derived from OJIP kinetics (Strasser et al., 2004). Red colour indicates the parameters recalculated by derivative-based time adjustment (Akinyemi et al., 2023).

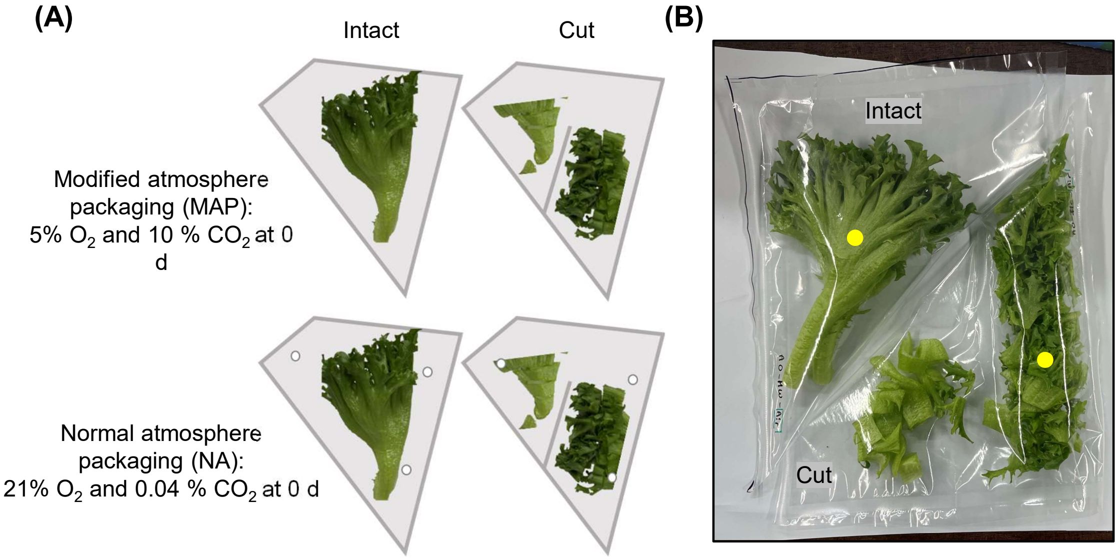

SUPPLEMENTAL FIGURE 1. Fresh-cut lettuce packages used in this study. (A) Schematic representation of modified atmosphere packaging (MAP) or normal atmosphere packaging (NA, puncture holes in packaging film) containing either two intact or cut lettuce leaves. (B) Representative photograph of intact or cut leaves in NA from ES1 on the day of processing (0 d), the yellow dot indicates the areas targeted for flash-induced (OJIP) chlorophyll fluorescence and data extraction following Pulse-Amplitude-Modulation (PAM) fluorometry. On intact leaves, the targeted area was consistently selected in the middle of the leaf on the abaxial side.

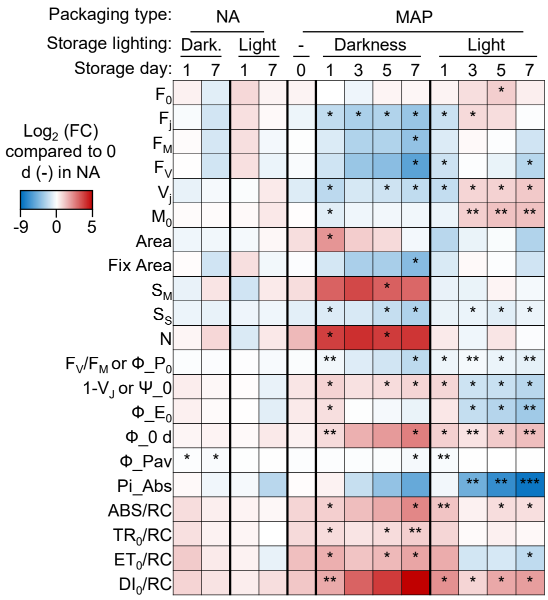

SUPPLEMENTAL FIGURE 2. Log2 of the average fold change of photosynthetic parameters inferred from OJIP kinetics of intact detached lettuce leaves (ES1) stored in NA or MAP just after packaging on day 0 (0 d) with no storage (-) and up to seven days of storage under darkness at 7°C or 30-52 µmol photons m^{-2} s^{-1} light at 10°C (7°C initial setting) and 50% RH normalized to the respective parameter value of leaves at 0 d (-) in NA.

Throughout storage, average gas compositions were 21% O_2 and 0.04% CO_2 in NA and varied from the initial 5% O_2 and 10% CO_2 in MAP as shown in Figure 1A, B. By 7 d in MAP, gas compositions were 3.6 ± 1.8% O_2 and 3.0 ± 1.2% CO_2 in darkness and 20.1 ± 0.6% O_2 and 0.0 ± 0.0% CO_2 in light.

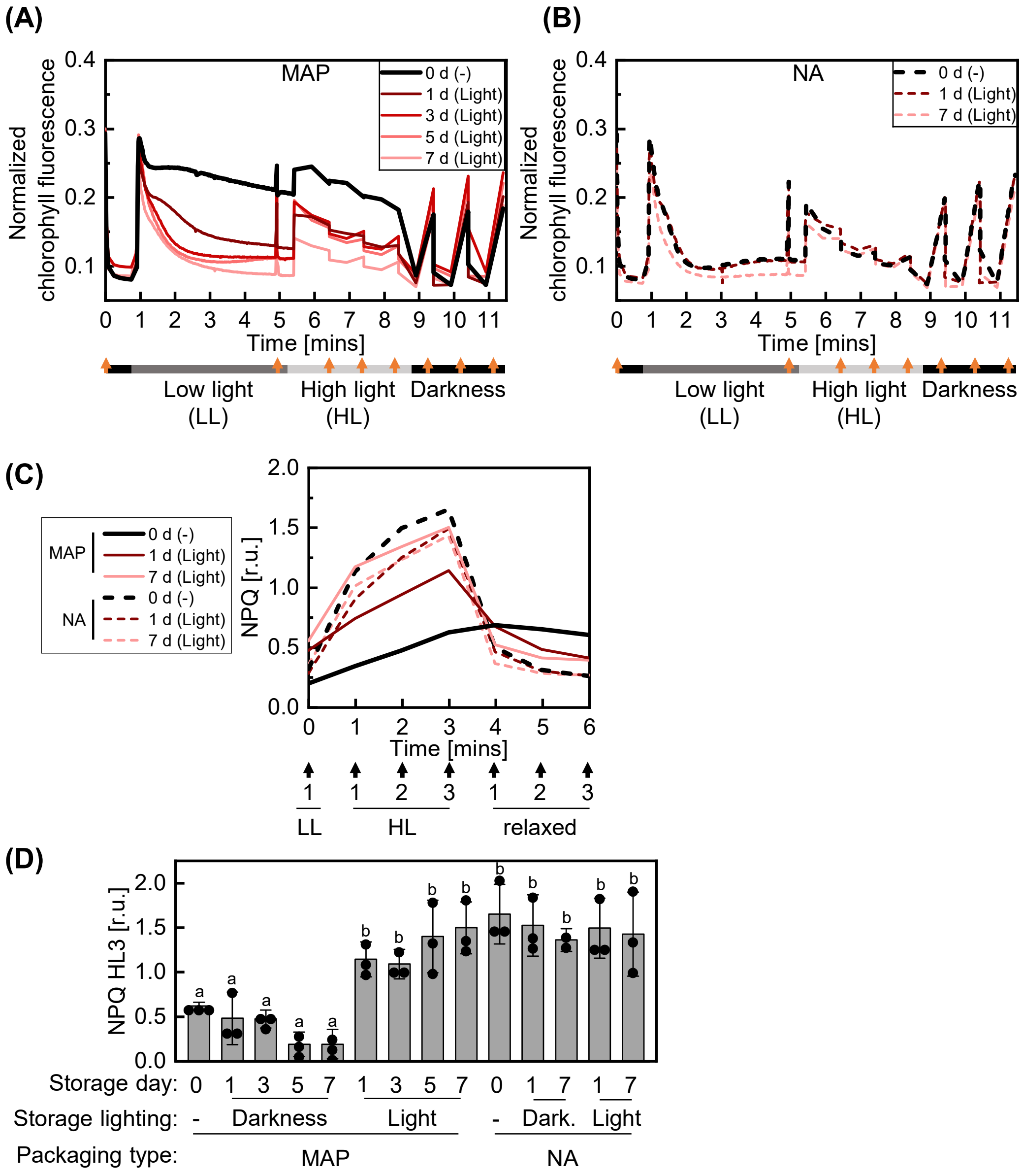

SUPPLEMENTAL FIGURE 3. PAM CF analysis in fresh-cut lettuce during storage in MAP or NA (A, B, accordingly). Chlorophyll *a* fluorescence measured by PAM fluorimetry under specific light/dark conditions and saturating light pulses (orange arrows) and (C) the corresponding NPQ buildup/relaxation curves from intact detached lettuce leaves packed in normal atmosphere (NA, puncture holes in packaging film) or modified atmosphere packaging (MAP) just after packaging on day 0 (0 d) with no storage (-) and up to 7 days of 30-52 μmol photons m^{-2} s^{-1} light at 10°C (7°C initial setting) and 50% RH. (D) Bar plot representation of NPQ following the third saturating flash under high light as shown in (C) and in Figure 4C following storage under darkness at 7°C. The data (ES1) represent the mean (A-C) or mean ± SD (D) across 2-3 time-independent experimental replicates and with 1 biological replicates each. Throughout storage, average gas compositions were 21% O_2 and 0.04% CO_2 in NA and varied from the initial 5% O_2 and 10% CO_2 in MAP as shown in Figure 1A, B. By 7 d in MAP, gas compositions were 3.6 ± 1.8% O_2 and 3.0 ± 1.2% CO_2 in darkness and 20.1 ± 0.6% O_2 and 0.0 ± 0.0% CO_2 in light.

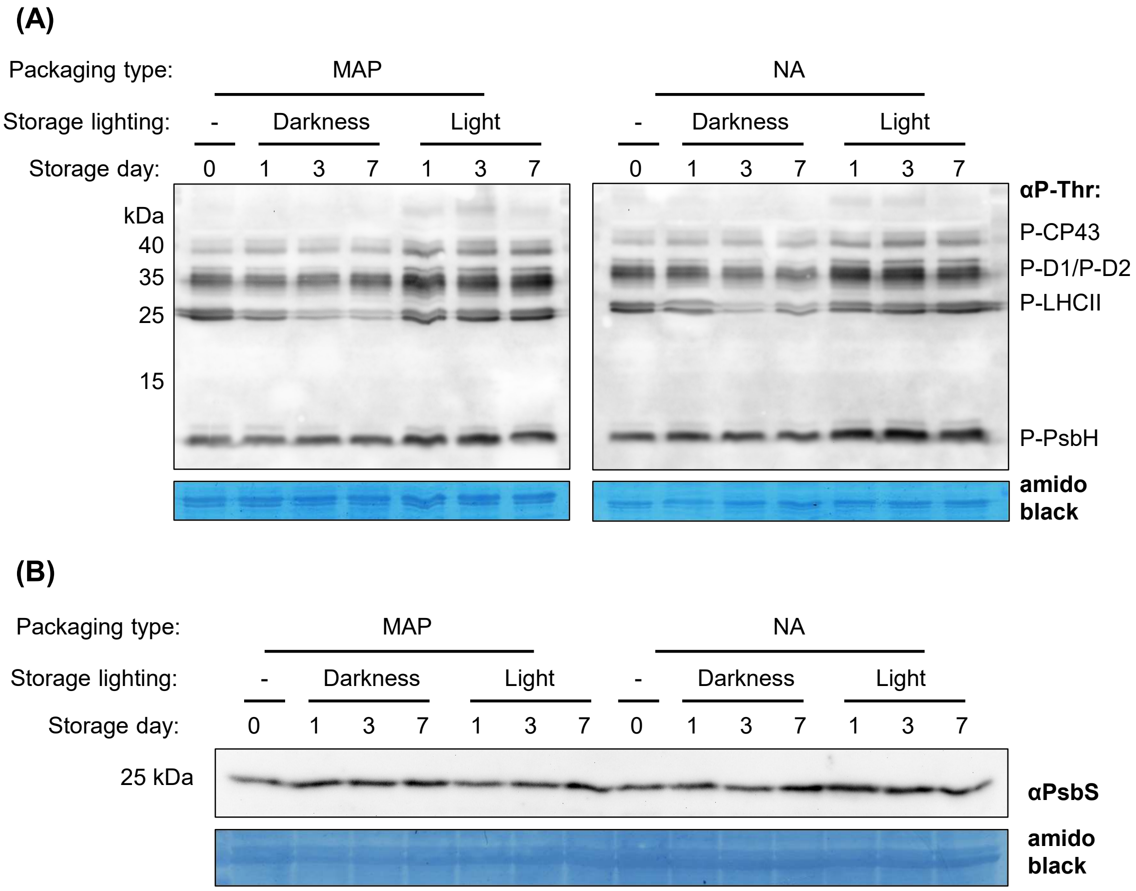

SUPPLEMENTAL FIGURE 4. Assessment of phosphorylation levels of photosynthetic complexes (A) and abundance of PsbS protein (B) in thylakoids isolated from detached lettuce leaves (ES2) packed in NA or MAP just after packaging on day 0 (0 d) with no storage (-) and up to seven days of darkness at 7°C or 40-54 µmol photons m^{-2} s^{-1} light at 10°C (7°C initial setting) and 50% RH. Amido black staining of LHCII antenna complexes is shown as the loading control.
